# New insights into acinic cell carcinoma of the breast: clinicopathology, origin of histology, molecular features, prognosis, and treatment

**DOI:** 10.3389/fonc.2024.1438179

**Published:** 2024-09-02

**Authors:** Yunjie Ge, Xianping Wei, Jing-Nan Liu, Ping-Li Sun, Hongwen Gao

**Affiliations:** ^1^ Department of Pathology, The Second Hospital of Jilin University, Changchun, Jilin, China; ^2^ Department of Clinical Research, The Second Hospital of Jilin University, Changchun, Jilin, China; ^3^ Department of Respiratory Medicine, The First Affiliated Hospital of Jilin University, Changchun, Jilin, China

**Keywords:** acinic cell carcinoma, clinicopathology, salivary gland-type tumor, triple-negative breast cancer, immunohistochemistry, molecular genetics

## Abstract

Acinic cell carcinoma (AciCC) of the breast is a rare malignant epithelial neoplasm, with approximately 60 cases reported in the literature. It predominantly affects women and exhibits significant histological heterogeneity. The diagnosis of breast AciCC is primarily based on the presence of eosinophilic and/or basophilic granular cytoplasm and markers of serous acinar differentiation. Despite being considered a low-grade variant of conventional triple-negative breast cancer (TNBC), over 25% of patients with breast AciCC have adverse clinical outcomes. Additionally, in early research, microglandular adenosis (MGA) and atypical MGA were considered potential precursors for various breast cancers, including intraductal carcinoma, invasive ductal carcinoma, adenoid cystic carcinoma, metaplastic carcinoma, and AciCC. Similarly, some studies have proposed that breast AciCC should be considered a type of carcinoma developing in MGA with acinic cell differentiation rather than a distinct entity. Therefore, the pathogenesis of breast AciCC has not yet been clarified. Moreover, to the best of our knowledge, the literature has not summarized the latest prognosis and treatment of breast AciCC. In this review, we synthesized the current literature and the latest developments, aiming at exploring the clinicopathology, histological origin, molecular features, prognosis, and treatment of breast AciCC from a novel perspective.

## Introduction

Acinic cell carcinoma (AciCC) of the breast was first reported in 1996 by Roncaroli et al. (1) and originally named breast acinar cell-like carcinoma that showed a predominantly solid pattern composed mostly of polygonal neoplastic cells with finely granular cytoplasm. The histological patterns of breast AciCC overlap with those of salivary AciCC, and the 5th World Health Organization classification of tumors of the breast (2) lists it as a rare and salivary gland-type tumor. However, studies have demonstrated that both have different genetic underpinnings (3, 4). Therefore, it seems that the naming and subtype classification of breast AciCC are controversial. Additionally, although breast AciCC is considered to have indolent biological behavior, the presence of high-grade histological morphology is not uncommon (5–7). Genetic studies have also revealed similarities between some breast AciCC and conventional aggressive triple-negative breast cancer (TNBC) (3, 8–11). Hence, the true nature of breast AciCC remains unclear. Moreover, due to the increase in recurrent, metastatic, and fatal cases (12–14), it is necessary to explore the prognostic features and treatment options for better patient management and treatment decision-making. This review summarizes the most recent research on breast AciCC, presenting new insights into the histopathological, immunohistochemical, and genetic features, pathogenesis, prognosis, and treatment of this rare entity.

## Clinical features

Breast AciCC has been confused with other types of breast cancer in the early literature. This review performed a clinicopathological analysis on 54 cases reported in the literature, excluding cases with an unclear diagnosis. We gave the total number of cases before analyzing each parameter because not all cases had complete clinicopathological data. Breast AciCC mostly affects women (53/54, 98.1%), with one 23-year-old man reported in 1998 (15). The age range in the series is 23–80 years, with a median age of 47.5 (39.8, 58.3) years and a mean age of 49.5 (14.5) years. Approximately 86.8% (33/38) of clinical manifestations are a palpable mass in the breast, especially in the upper outer quadrant (16/23, 69.6%) of the breast. Clinical laboratory tests are usually normal. Ultrasound typically reveals a hypoechoic mass, while mammography usually shows an ill-defined, solid, cystic, or lightly lobulated mass, with or without microcalcification. **Table 1** presents a summary of the clinical and histopathological parameters of breast AciCC in the previous literature.

**Table 1:** Clinicopathological features of breast AciCC in the previous literature.

Clinical and histopathological parameters	N	%
Sex (N = 54)		
Male	1	1.9
Female	53	98.1
Age (year) (N = 54)		
Range	23–80	
Median (P25, P75)	47.5 (39.8, 58.3)	
Mean (SD)	49.5 (14.5)	
Clinical symptom or sign (N = 38)		
A palpable mass	33	86.8
A nonpalpable mass	1	2.6
Enlarged axillary lymph node	2	5.3
Skin retraction/depression of the nipple	2	5.3
Quadrants of the breast (N = 23)		
Upper outer quadrant	16	69.6
Upper inner quadrant	3	13.0
Lower outer quadrant	2	8.7
Above the nipple or peri-areolar	2	8.7
Tumor size (mm) (N = 50)		
Range	10–71	
Median (P25, P75)	29.0 (20.0, 41.3)	
Mean (SD)	31.0 (14.7)	
Growth patterns (N = 50)		
Acinar/glandular/tubular/micro-glandular	44	88.0 (44/50)
Solid nests/cords/trabecular	29	58.0 (29/50)
Single-cell infiltration	8	16.0 (8/50)
Cystic/microcystic	5	10.0 (5/50)
Cribriform/pseudo-lobular	3	6.0 (3/50)
Papillary/micropapillary	3	6.0 (3/50)
Lymph node status (N = 33)		
Positive	9	27.3
Negative	24	72.7
Surgery (N = 41)		
BCS	3	7.3
BCS + ALDN/SLDN	11	26.8
MRM + ALDN/SLDN	17	41.5
Partial/total mastectomy + ALND/SLDN	9	22.0
WLE + SLDN	1	2.4
Therapeutic regimen (N = 22)		
Neo-CT/CT/ HT/RT	9	40.9
CT + RT	7	31.8
Neo-CT + CT/HT/RT	4	18.2
Neo-CT/CT + HT + RT	2	9.1
Follow-up (months) (N = 43)		
Range	3–184.8	
Median (P25, P75)	21.0 (12.0, 48.0)	
Mean (SD)	37.4 (39.9)	
Outcome (N = 43)		
Recurrences/metastases/death	11	25.6
NED	32	74.4

P25, the 25th percentile; P75, the 75th percentile; SD, standard deviation; BSC, breast-conserving surgery; ALND, axillary lymph node dissection; SLND, sentinel lymph node dissection; MRM, modified radical mastectomy; WLE, wide local excision; Neo-CT, neoadjuvant chemotherapy; CT, chemotherapy; HT, hormone therapy; RT, radiation therapy; NED, no evidence of disease.

## Pathological features

**Figure 1:**
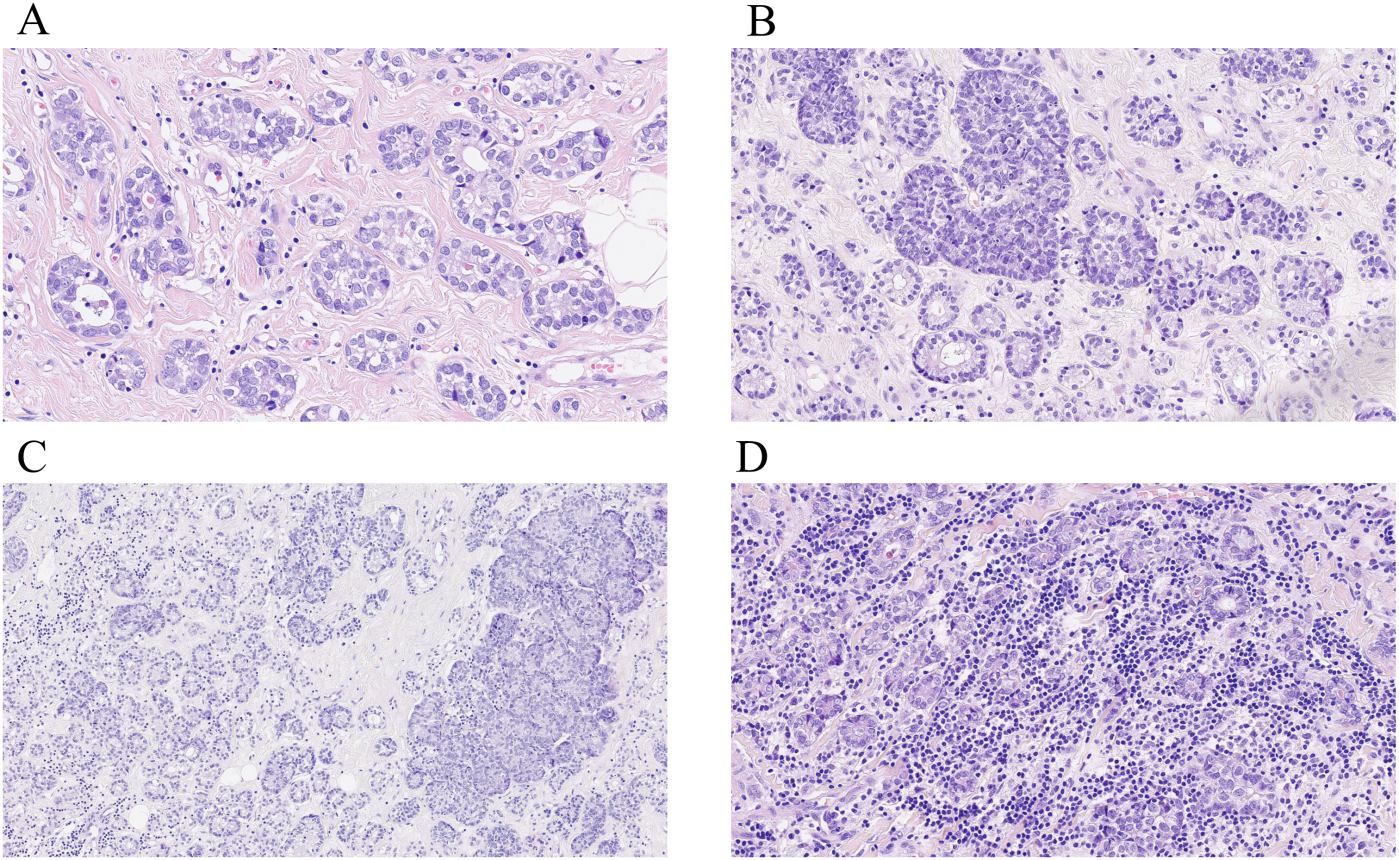
Histological characteristics of breast AciCC. Microscopically, the predominant morphologic patterns of breast AciCC are round to irregularly shaped acinars (**A**, H&E, ×200) and solid patterns (**B**, H&E, ×200). Both morphological structures usually merge together (**C**, H&E, ×200). Stromal tumor-infiltrating lymphocytes can be observed (**D**, H&E, ×200).

On gross examination, breast AciCC usually shows a well-defined, non-encapsulated mass (10–71 mm), with a median size of 29.0 (20.0, 41.3) mm and a mean size of 31.0 (14.7) mm. In 50 cases of breast AciCC, data on morphological features were available. Histologically, breast AciCC grows infiltratively and usually presents significant morphological heterogeneity. The predominant morphologic patterns are round to irregularly shaped acinar, glandular, tubular, or microglandular adenosis (MGA)-like structures (44/50, 88.0%) (**Figure 1A**) and solid patterns (29/50, 58.0%) (**Figure 1B**). Both morphological structures usually merge together (**Figure 1C**). Stromal tumor-infiltrating lymphocytes can be observed (**Figure 1D**). The acinic or glandular epithelia are lined by single to several layers of neoplastic cells (16). Neoplastic cells in solid patterns usually arrange in irregular solid nests with or without necrosis, cords, trabecular, labyrinthine-like patterns, or a single-cell infiltrative pattern (11–13, 17, 18). In a recent case, the tumor showed an invasive lesion that grew in an extensively solid and MGA-like pattern with high mitosis counts spreading continuously over a 159 × 121-mm area (19). Cytologically, the neoplastic cells are round to polygonal in shape with eosinophilic and/or basophilic granular cytoplasm, round or oval nuclei, and fine or rough chromatin. PASD (periodic acid–Schiff–diastase) (26/26, 100%) shows positive for intracytoplasmic eosinophilic granules. The neoplastic cells sometimes show clear cytoplasm. Nucleoli are often observed (7, 20, 21). The mitotic counts and cellular atypia are higher in solid areas compared to those in neoplastic glandular or acinic areas.

## Immunohistochemical features

**Figure 2:**
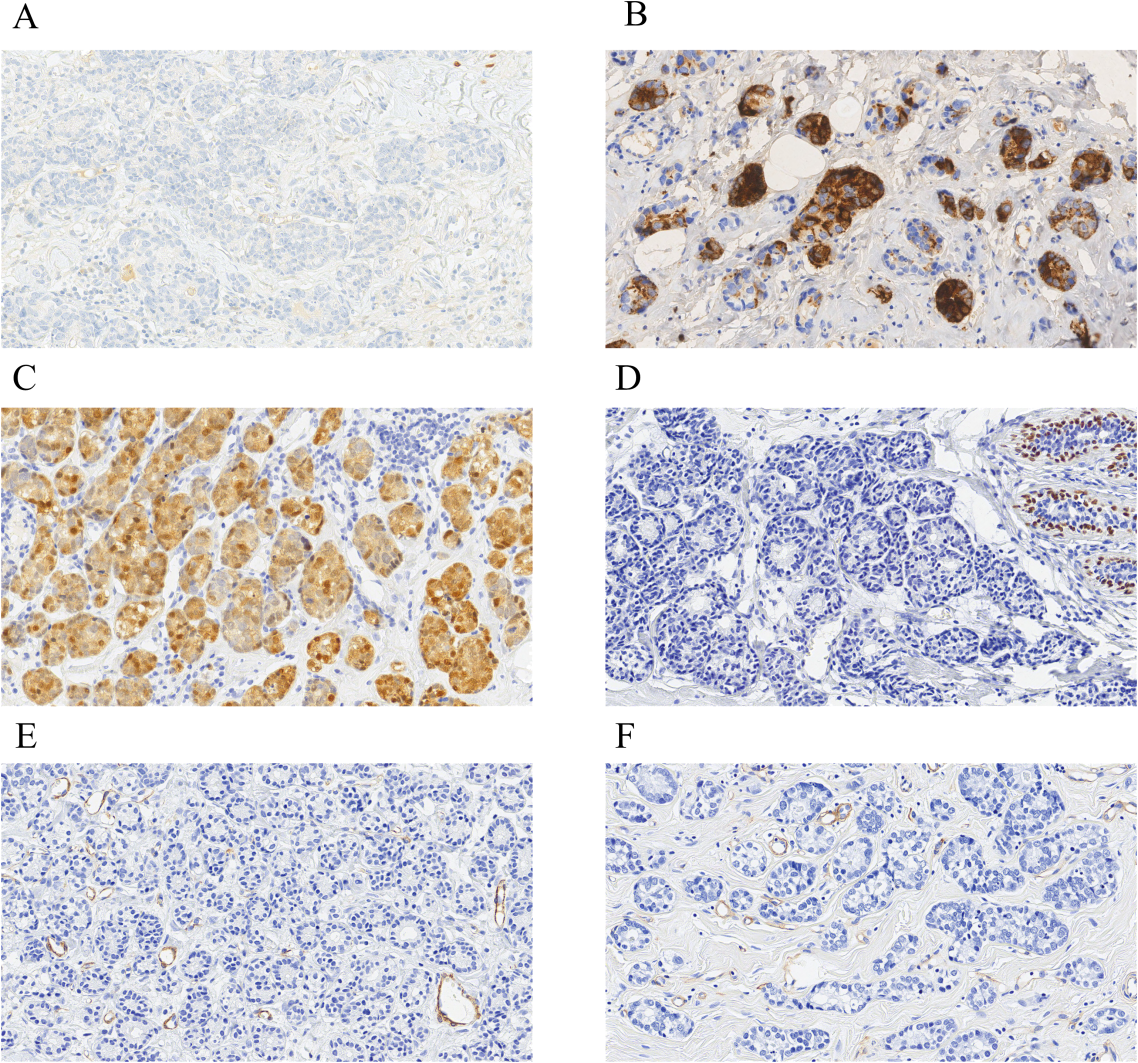
Immunohistochemical characteristics of breast AciCC. Breast AciCC is usually negative for ER (**A**, EnVision, ×200), PR, and HER2 and positive for LYS (**B**, EnVision, ×200), and S100 (**C**, EnVision, ×200). Because of the lack of peripheral myoepithelial cells, breast AciCC is negative for p63 (**D**, EnVision, ×200) and SMA (**E**, EnVision, ×200). Collagen Type IV (**F**, EnVision, ×200) staining shows the absence of basement membrane around glands and highlights the capillaries.

We obtained immunohistochemical data from 54 cases (some staining data were incomplete). From 37 cases, we acquired staining data of the estrogen receptor (ER) (**Figure 2A**), progesterone receptor (PR), and human epidermal growth factor receptor 2 (HER2). Approximately 73.0% (27/37) of breast AciCC showed a triple-negative immunophenotype. A detailed immunohistochemical expression of breast AciCC in the previous literature is summarized in **Table 2**. Although most immunohistochemical markers of breast cancer are not specific to breast AciCC, markers of serous acinar differentiation, such as lysozyme (LYS) (95.2%) (**Figure 2B**), amylase (AMY) (88.9%), α1-antichymotrypsin (95.2%) (AACT), and α1-antitrypsin (AAT) (60.0%), are helpful in differential diagnosis between breast AciCC and other epithelial neoplasms of the breast. The expression of low-molecular weight cytokeratin (CK-LMW) (100%), epithelial membrane antigen (EMA) (100%), and S100 (91.7%) (**Figure 2C**) is positive. Gross cystic disease fluid protein 15 (GCDFP-15) (66.7%), cytokeratin 7 (CK7) (88.9%), and E-cadherin (85.7%) show variable positivity in different cases. Breast AciCC is negative for myoepithelial markers, such as p63 (0%) (**Figure 2D**), smooth muscle actin (SMA) (0%) (**Figure 2E**), and calponin (0%). Additionally, because there is no peripheral basal lamina in breast AciCC, the expression of laminin and collagen type IV (**Figure 2F**) is absent. However, they can show positive expression for MGA or MGA-like components that coexist with breast AciCC (11, 19).

**Table 2:** Immunohistochemical expression of breast AciCC in the previous literature.

Immunohistochemical markers	% Positivity
ER	10.9 (5/46)
PR	15.6 (7/45)
AR	18.2 (2/11)
HER2	5.4 (2/37)
Triple-negative immunophenotype	73.0 (27/37)
LYS	95.2 (40/42)
AMY	88.9 (16/18)
AACT	95.2 (20/21)
AAT	60.0 (6/10)
CK-LMW or pan-CK	100 (6/6)
Vimentin	20.0 (1/5)
EMA	100 (26/26)
GCDFP-15	66.7 (10/15)
CK7	88.9 (8/9)
GATA3	66.7 (4/6)
S100	91.7 (33/36)
E-cadherin	85.7 (6/7)
SMA	0 (0/16)
p63	0 (0/14)
calponin	0 (0/3)
Collagen type IV	25.0 (3/12)
Laminin	25.0 (2/8)
CgA	7.1 (1/14)
Syn	0 (0/9)

ER, estrogen receptor; PR, progesterone receptor; AR, androgen receptor; HER2, human epidermal growth factor receptor 2; LYS, lysozyme; AMY, amylase; AACT, α1-antichymotrypsin; AAT, α1-antitrypsin; CK-LMW, low-molecular weight cytokeratin; pan-CK, pan cytokeratin; EMA, epithelial membrane antigen; GCDFP-15, gross cystic disease fluid protein 15; CK7, cytokeratin 7; GATA3, GATA-binding protein 3; SMA, smooth muscle actin; CgA, chromogranin A; Syn, synaptophysin.

## Origin of histology

Breast glands are tubulo-acinar exocrine glands, and breast AciCC can show salivary gland acinar differentiation, which is considered to be one of the important histologic bases for the origin of breast AciCC (22). These salivary gland acinar differentiation cells have the characteristics of serous acinar cells, such as eosinophilic or basophilic cytoplasm, and the presence of zymogen granular material stained positive for PASD. Although sharing embryologic and morphologic similarities, breast AciCC and salivary AciCC have different molecular features. Salvatore Piscuoglio et al. (3) performed sanger sequencing on the entire coding region of the *TP53* and *PIK3CA* hotspot mutation sites of 10 breast and 20 salivary AciCC. This study found that *TP53* (8/10, 80%) and *PIK3CA* (1/10, 10%) mutations were present in breast AciCC but not in salivary AciCC. Furthermore, recurrent genomic rearrangement t(4; 9) (q13; q31) in salivary AciCC specifically increased the level of the nuclear transcription factor NR4A3, detectable by the immunohistochemical marker NR4A3. However, this marker is absent in breast AciCC (4, 23).

Aside from salivary AciCC, the morphologic patterns of breast AciCC, especially neoplastic acinic structures, are also similar to MGA. Both have a triple-negative immunophenotype and express LYS, S100 (8). MGA is defined as a haphazard proliferation of small glands, consisting of a single layer of epithelial cells without an accompanying myoepithelial cell layer. When the glands of MGA merge together into solid or cribriform nests with cellar atypia, it is referred to as atypical MGA (24). One of the differential diagnoses between MGA/atypical MGA and breast AciCC is that the latter lacks a basement membrane. However, several studies have observed morphological and immunohistochemical evidence of the transition from typical MGA to atypical MGA and then to AciCC (19, 21). Later genetic studies supported the contention that MGA, atypical MGA, and breast AciCC may be part of the same spectrum of lesions harboring frequent *TP53* somatic mutations and represent low-grade forms of TNBC with the potential to progress to high-grade TNBC (8). Conlon et al. advocated that MGA-like areas at the periphery of breast AciCC should be considered part of the carcinomatous process and re-excised if it extends to the initial surgical margins (11).

Previous studies show that MGA and atypical MGA may constitute non-obligate precursors of many types of breast cancer, including intraductal carcinoma, invasive ductal carcinoma, adenoid cystic carcinoma, metaplastic carcinoma, and AciCC (25–28). Although the morphology of the associated carcinomas varies, they share similar immunophenotypes with MGA (S−100 positive expression, ER, PR, and HER2 negative expression), and the transition from MGA to atypical MGA and then to the associated carcinoma is observed (28). MGA and its associated carcinomas share similar molecular alterations; however, pure MGA shows different molecular alterations from MGA with associated carcinoma, despite sharing similar histological patterns (8, 29). Additionally, researchers genetically analyzed four breast AciCC mixed with high-grade carcinoma, including three cases of invasive ductal carcinoma (no special type) and one case of metaplastic carcinoma focusing on the acinic and high-grade non-acinic components. The result revealed that identical somatic mutations were identified in different components of two cases suggesting the clonal relatedness of acinic and high-grade non-acinic components (10). Therefore, the high-grade non-acinic components in mixed breast AciCC may also be closely related to MGA. However, because of the limited number of reported cases and related genetic studies, it is essential to accumulate additional cases for further comprehensive investigation.

## Molecular features

A review of prior reports demonstrates that *TP53* is the most commonly mutated gene in breast AciCC cases (8, 12). Molecular studies showed that breast AciCC displays similar complex patterns of copy number (CN) alterations and mutations of genes, such as *TP53*, *PIK3CA*, *MTOR*, *CTNNB1*, *BRCA1*, *ERBB4*, *ERBB3*, *INPP4B*, and *FGFR2*, that are akin to conventional high-grade TNBC (8, 9). A breast AciCC case (30) was diagnosed in our hospital, and the tumor was microscopically composed of a classical acinic component and a high-grade solid component. The histological transition from the acinic area to the solid region was observed. We performed next-generation sequencing on both components separately, targeting all the exons of 769 cancer-related genes. The results revealed that 10 (10/23, 43.5%) variants were identical in both components, including the mutations of *TP53*, *LMO1*, *MDC1*, *MSH3*, *KMT2D*, and *CCND3*, as well as the CN gains of *CCND1*, *FGFR2*, *MYC*, and *IDH1*. Remarkably, each of these shared variants was more complex in the high-grade lesion. Furthermore, *KMT2C* (c.161 + 1G > A), *ALOX12B*, *KDM5A*, *PIK3CD*, and *POLE* mutations were identified in the classical component. *KMT2C* (c.250 + 1G > A) and *PAK5* mutations; CN loss of *CDH1*, *CHEK1*, and *MLH1*; and CN gains of *CDK6*, *HGF*, and *FOXP1* were identified in the high-grade component.

A whole-exome and RNA-sequencing analysis of three breast AciCC cases (9) detected *TP53* hotspot mutations in the first two cases. The *TC2N*–*FBLN5* intra-chromosomal fusion gene and focal amplification of 12q14.3–12q21.1 of *MDM2*, *HMGA2*, *WIF1*, *FRS2*, and *PTPRB* were identified in one case. Another case detected a focal amplification in 20p12.3 of *PCNA* and a somatic homozygous deletion in 17q21.31 of *BRCA1*. In the third case (*TP53* mutation wild type), a pathogenic *MLH1* germline mutation (c.790 + 2dupT) and a clonal hotspot mutation in *CTNNB1* (c.1004A>T) were found. The abnormalities of *BRCA1* and *MLH1* are important factors in homologous recombination deficiency and high microsatellite instability, respectively. Therefore, they were detected in breast AciCC (9, 31) providing a theoretical foundation for the molecularly targeted therapy for breast AciCC. In a recent study (12), two breast AciCC exhibited the same *MED12* mutation (NM_005120.2; exon 27, c.3817G>T; p.A1273S) with similar mutation abundance. Both tumors displayed the morphological features characterized by intricate burrowing labyrinthine networks, or “hand-holding-hand” patterns. This may suggest a correlation between molecular alterations and morphology in breast AciCC.

## Prognosis

Survival data are available for 43 breast AciCC. The follow-up ranged from 3 to 184.8 months, with a median of 21.0 (12.0, 48.0) months and a mean of 37.4 (39.9) months. One (1/43, 2.3%) patient died of a primary tumor, five patients (5/43, 11.6%) developed recurrence, and five patients (5/43, 11.6%) suffered from bone, liver, lung, or peritoneal metastases (two patients later died of liver or peritoneal metastases). Eight of the 11 cases (8/11, 72.7%) had lymph node metastases or/and consisted of high-grade components in morphology (10–13, 17, 18, 32–34). Hence, periodic review and follow-up of patients are essential, especially for patients with breast AciCC mixed with high-grade components or lymph node metastases.

TNBC is a histologically heterogeneous tumor (35). Most TNBCs are high-grade invasive breast carcinomas with aggressive clinical course and adverse outcomes, and studies have shown that TNBC patients have a shorter disease-free survival and overall survival time compared to non-TNBC patients (36–39). Qiu et al. (39) revealed that the rate of recurrence and metastasis in TNBC patients was 27.95%, compared to 13.38% in non-TNBC patients. Studies have also demonstrated that histologically special types of TNBC, such as adenoid cystic carcinoma, medullary carcinoma, and apocrine carcinoma, have a better prognosis than invasive ductal carcinoma not otherwise specified type (40, 41). However, studies on the prognosis of breast AciCC are limited (13). In the review, follow-up information was available for 22 of the 27 breast cancers that showed a triple-negative immunophenotype. The median follow-up time was 24.0 (14.8, 61.2) months. Of the 22 patients, 5 (5/22, 22.7%) had adverse prognosis (one patient died; threee patients developed recurrence; and one patient suffered bone metastases).

Lymph node status represents a significant prognostic factor for breast cancer patients. This review gathered information on lymph node dissection from 33 breast AciCC patients, revealing that nine patients (9/33, 27.3%) had lymph node metastases. Studies have shown that TNBC has higher rates of lymph node metastasis (30%–50%) (42, 43), compared to non-TNBC. However, TNBC is not more likely to have involved nodes than non-TNBC in other studies (44, 45). We obtained lymph node data from 21 out of the 27 breast AciCC cases exhibiting a triple-negative immunophenotype. Among these, the lymph node metastasis rate was 23.8% (5/21).

## Treatment

Similar to TNBC, neoadjuvant therapy, surgery, adjuvant therapy, and radiotherapy are basic therapy regimens for breast AciCC. We obtained information about the surgical methods from 41 patients with breast AciCC. The most common method was modified radical mastectomy (MRM) combined with axillary lymph node dissection (ALND) or sentinel lymph node dissection (SLND) (17/41, 41.5%). According to the 2024 NCCN Breast Cancer Guidelines, for TNBC with recurrence unresectable, or stage IV disease, when the combined positive score (CPS) of PD-L1 ≥10, PD-1 inhibitor pembrolizumab combined with chemotherapy can be used for first-line therapy, regardless of germline *BRCA* mutation status. In one breast AciCC (46), a PD-L1 immunohistochemical test was performed (CPS = 3). The patient received chemotherapy and pembrolizumab immunotherapy, and she remained symptom-free for 14 months after surgery. For breast cancer patients with germline *BRCA1/2* mutation, the addition of 1 year of poly (ADP-ribose) polymerase (PARP) inhibitor olaparib after completion of adjuvant chemotherapy is prognostically beneficial. There have been two breast AciCC patients with germline *BRCA1* mutations (7, 31), but these patients did not receive PARP inhibitor therapy. A breast AciCC in a female patient without *BRCA1*/2 mutations received basic therapy, followed by olaparib adjuvant systemic therapy, and she proceeded with contralateral prophylactic mastectomy. Four years after surgery, there was no sign of a recurrence (47).

## Conclusion

Overall, we summarized the clinicopathological features and new research developments of breast AciCC. Morphologically, breast AciCC has significant heterogeneity. The predominant patterns of breast AciCC include round to irregularly shaped acinars and solid nests. Additionally, solid cords, trabecular, labyrinthine-like patterns, and a single-cell infiltrative pattern can also be observed. Breast AciCC shares morphological similarities with salivary AciCC, but they exhibit distinct molecular features. MGA and atypical MGA may represent one of the non-obligate precursor lesions for breast AciCC. Breast AciCC displays unique biological characteristics. Overall, breast AciCC has a lower aggressive potential than conventional TNBC, but it is not as indolent as other low-grade TNBC. The presence of lymph node metastases or high-grade components in breast AciCC is indicative of an unfavorable prognosis. The histological transition and identical genetic alternations of the classical acinic component and high-grade non-acinic component have been observed in the same breast AciCC, and genomic features resembling conventional TNBC have been identified in breast AciCC cases, indicating that breast AciCC has the potential to transform or progress to high-grade carcinoma. Furthermore, homologous recombination deficiency and high microsatellite instability can occur in breast AciCC providing a theoretical foundation for molecularly targeted therapy.
